# Age Structural Transitions and Copayment Policy Effectiveness: Evidence from Taiwan’s National Health Insurance System

**DOI:** 10.3390/ijerph17124183

**Published:** 2020-06-12

**Authors:** Ya-Ling Lin, Wen-Yi Chen, Shwn-Huey Shieh

**Affiliations:** 1 Department of Public Health, China Medical University, 91 Hsueh-Shih Road, Taichung 40402, Taiwan; yaling80372@gmail.com; 2 Department of Nursing, Taichung Hospital, Ministry of Health Welfare, 199, Sec. 1, Sanmin Road, Taichung 40343, Taiwan; 3 Department of Senior Citizen Service Management, National Taichung University of Science and Technology, 193, Sec. 1, Sanmin Road, Taichung 40343, Taiwan; 4 Department of Health Services Administration, China Medical University, 91 Hsueh-Shih Road, Taichung 40402, Taiwan; shshieh@mail.cmu.edu.tw

**Keywords:** copayment policy, age structural transitions, population ageing, National Health Insurance, policy effectiveness

## Abstract

Background: Population ageing is a worldwide phenomenon that could influence health policy effectiveness. This research explores the impact of age structural transitions on copayment policy responses under Taiwan’s National Health Insurance (NHI) system. Methods: The time-varying parameter vector autoregressive model was applied to create two measures of the copayment policy effectiveness, and multiple linear regression models were used to verify the nonlinear effect of age structural transitions on copayment policy responses. Results: Our results show that copayment policy effectiveness (in terms of the negative response of medical center outpatient visits to upward adjustments in copayment) is positively correlated with the proportions of the population in two older age groups (aged 55–64 and ≥ 65) and children (age < 15), but negatively correlated with the proportion of the population that makes up most of the workforce (aged 15‒54). These tendencies of age distribution, which influence the responses of medical center outpatient visits to copayment policy changes, predict that copayment policy may have a greater influence on medical center outpatient utilization in an ageing society. Conclusions: Policymakers should be concerned about the adverse effects of copayment adjustments on the elderly, such as an increasing financial burden and the effect of pricing some elderly patients out of Taiwan’s NHI system.

## 1. Introduction

### 1.1. Background

Taiwan’s National Health Insurance (NHI) system is a government-implemented social insurance program providing universal healthcare coverage (based on pay-as-you-go principles) to all residents in Taiwan. This system has encountered tremendous financial difficulties over the past two decades due to the nature of publicly financed healthcare systems [1]. A sizeable proportion of total medical care expenditures was spent on outpatient care services in Taiwan’s NHI system since it was implemented in 1995. In 2016, approximately 70% of total medical expenditures were for outpatient care services, with 58.25% of this going to medical centers (23.51%), regional hospitals (23.84%) and district hospitals (10.90%) [2]. These three levels of hospitals have different responsibilities within Taiwan’s NHI system [3,4]. In particular, medical centers are oriented to deal with the most complex diseases, and to support teaching and research in clinical practices. District hospitals and regional hospitals are responsible for secondary and tertiary care, respectively. In addition to hospitals, local clinics in Taiwan are built to deal with primary care. Over 80% of children (age < 15) receive their outpatient care from the local clinics, and the elderly (aged 65 and older) and youth (aged 15‒24) contribute the largest (approximately 36.88‒38.90%) and smallest shares (approximately 3.03‒3.83%) of total outpatient care visits, respectively, to medical centers, regional hospitals, and district hospitals [2,5]. It is important to note that the reimbursement payments per outpatient visit to district hospitals (NT$ 1770 or about USD 59), regional hospitals (NT$ 2445 or about USD 82) and medical centers (NT$ 3261 or about USD 109) were 2.37‒4.36 times higher than those made to local clinics (NT$ 748 or about USD 25) in 2016 [2]. With the ageing population in Taiwan, the referral system becomes an important issue to consider, with regard to the financial difficulties of Taiwan’s NHI system.

In order to strengthen the referral system in a way that could reduce outpatient care expenditure in hospitals (particularly in medical centers), Taiwan’s Ministry of Health and Welfare (MOHW) has adjusted copayments for outpatient care multiple times since 1995 [6,7]. During our study period, from January 1998 to December 2015, Taiwan’s MOHW revised the copayment policies for outpatient care utilization in 1999, 2002 and 2005 [6,7]. The 1999 co-payment policy introduced co-payments (up to NT$ 100 or about USD 3.33) for prescription drugs, and an additional NT$ 50 (about USD 1.67) copayment fee per visit for excessive outpatient visits (more than 24 visits per year), while the 2002 copayment policy further increased the copayment fees for outpatient care visits in medical centers and regional hospital from NT$ 150 (about USD 5) to NT$ 210 (about USD 7), and from NT$ 100 (about USD 3.33) to NT$ 140 (about USD 4.67), respectively. In 2005, the design of the copayment policy for outpatient care utilization under Taiwan’s NHI system offered a price-differentiating mechanism, leading patients to select healthcare providers from a local clinic for their first outpatient visit, instead of going directly to a hospital. This price-differentiating mechanism was designed to raise the copayment fees for outpatient visits at medical centers, regional hospitals and district hospitals to NT$ 360 (about USD 9), NT$ 240 (about USD 8) and NT$ 80 (about USD 2.67), from NT$ 210 (about USD 7), NT$ 140 (about USD 4.67) and NT$ 50 (about USD 1.67), respectively, if the patient was not referred from a local clinic. Those who were referred to hospitals from a local clinic paid copayment fees at the pre-2005 rates at hospitals (i.e., medical centers, regional and district hospitals) [6,7]. 

The design of the price-differentiating mechanism, intended to change patients’ behavior when seeking outpatient care, was also used for the new copayment policy for outpatient care (effective April 15, 2017). Specifically, the new co-payment fees for direct visits to medical centers, regional hospitals and district hospitals are 2.47, 2.40 and 1.6 times higher, respectively, than for those with referrals, and this policy also creates a price gap of NT$ 250 (or about USD 8.33) per outpatient visit, between the price for direct visits and the price for those with referrals to medical centers, an amount much higher than the price for regional hospitals (NT$ 140 or about USD 4.67) and district hospitals (NT$ 30 or about USD 1.00) [6]. The effectiveness of the new copayment policy in reducing outpatient care expenditure depends on how patient behavior in seeking outpatient care responds to the changes in medical center outpatient visit prices under Taiwan’s NHI system. In this study, we applied the time-varying parameter vector autoregressive model (developed by Nakajima [8]) and multiple linear regression models, in order to specifically explore the impact of age structural transitions on copayment policy effectiveness in medical center outpatient care. This was done because the recent nonreferral copayment policy under Taiwan’s NHI system focused on decreasing medical center outpatient care utilization, due to its significant contribution to total outpatient care expenditure, and a substantial reimbursement-payment-per-visit gap between medical center and clinic outpatient visits [2,6]. In addition, we chose Taiwan as our study area for examining the impact of age structural transitions on copayment policy effectiveness because the Taiwanese population is now experiencing rapid population ageing, and it is predicted that it will take only 33 years to move from an ageing to a hyper-aged society [9]. Since our time-varying parameter vector autoregressive model is capable of dealing with the changes in outpatient care utilization related to the changes in copayment policies made during our study period, the results generated in this study can provide insight into the influence of population ageing on the healthcare system.

### 1.2. Literature Reviews

Recent studies on the effectiveness of copayment policy in curbing healthcare expenditure suggest a significantly negative relationship between copayment and healthcare utilization. For example, Kill and Houlberg conducted a systematic literature review investigating the influence of copayments on healthcare demand. Their review identified 47 eligible studies on the behavioral effects of copayment, and the majority of their reviewed studies suggested that copayments reduce outpatient care utilization [10]. Recent systematic literature reviews, such as Kolasa and Kowalczyk [11] and Sensharma and Yabroff [12], investigating the relationships between patient cost-sharing of prescription drugs, healthcare utilization and health outcomes, suggested that an increase in patient cost-sharing of prescription drugs not only decreases prescription drug utilization, but also increases the risk of worsening health outcomes (in terms of deteriorating adherence to prescription drugs), and increases the demand for healthcare services, such as the emergency room, outpatient services and inpatient care services. Another strand of the literature, on the effect of user fee (consisting of copayment and cost-sharing) changes on healthcare utilization, applied a difference-in-differences (DID) regression model to evaluate the effect of user fees on the utilization of various healthcare services. In general, a negative association between user fees and healthcare utilization was found for inpatient care services [13,14,15], outpatient care services [14,16,17,18,19,20,21,22], long-term care utilization [23,24], psychiatric care services [25,26], rehabilitation care services [27] and prescription drug usage [28,29,30].

Taiwan’s MOHW has modified the copayment policies for outpatient care utilization many times since Taiwan’s NHI program was implemented in 1995 [6,7]. Many studies have focused on the response of outpatient care utilization to the user fee change under Taiwan’s NHI system. For instance, Yang, Tsai and Tien investigated the effect of persistent behavior and cost-sharing policy on outpatient care utilization by the elderly in Taiwan, and the results obtained from their dynamic panel count data model indicated that the elderly Taiwanese population is more price-sensitive in the long run than in the short run, and that therefore the effects of copayment intervention on elderly outpatient care utilization would be more effective in the long run than in the short run [31]. Liu, Hsu and Huang adopted a conventional time series model to examine the determinants of health expenditure in Taiwan; their results show that upward adjustments in copayments for healthcare services covered by the NHI in 1999 had a significant impact, in terms of curbing healthcare expenditure [32]. A significantly negative relationship between the 1999 copayment adjustments and outpatient care utilization was also found in Huang and Tung’s study [33]. Additionally, Chen, Schafheutle and Noyce [34], and Chen, Bermell and McMullen [7], evaluated the impact of nonreferral outpatient copayments on outpatient care utilization (effective July 15, 2005), based on the estimation of the segment time series model and the sample selection model for count data, respectively. The results generated by these two studies also suggest a negative relationship between copayments and outpatient care utilization. 

Although the empirical findings from the aforementioned studies suggest that copayment policy may be effective in decreasing healthcare utilization, the link between age structural transitions and copayment policy effectiveness remains unclear in the literature on health economics and policy. This is becoming a particularly important issue as population ageing becomes a worldwide phenomenon. In fact, recent studies, such as those by Chen [35] and Imam [36], have begun to establish the influence of age structural transitions on monetary policy, in the form of making adjustments to the money supply by manipulating interest rates in order to achieve relevant economic objectives, such as economic growth or the reduction of unemployment rates. In order to make up for the deficit in studies on how population ageing impacts copayment policy effectiveness, we closely followed the empirical procedures suggested by Chen [35] to examine the impact of age structural transitions on copayment policy effectiveness. Accordingly, a time-varying parameter vector autoregressive model was employed to create time-varying impulse responses of outpatient care utilization to copayment adjustments, and this allowed us to construct two measures of copayment policy effectiveness (i.e., maximum and accumulative maximum responses of medical center outpatient visits to upward adjustments in copayments). 

## 2. Materials and Methods 

### 2.1. Time-Varying Parameter Vector Autoregressive Model 

Since the patient has complete freedom to choose healthcare providers, and the recent nonreferral copayment policy (effective April 15, 2017) focuses on decreasing outpatient visits to medical centers, our investigation into the impact of age structural transitions on the response of copayment change to outpatient utilization is based on the demand for medical center outpatient visits, as follows:

(1) $$q_{t}^{m}=f(p_{t}^{m},p_{t}^{r},p_{t}^{d},p_{t}^{c},W_{t})+\zeta_{t}$$

where $q_{t}^{m}$ denotes outpatient visits per capita in medical centers, and $p_{t}^{m},p_{t}^{r},p_{t}^{d}$ and $p_{t}^{c}$ represent copayments per outpatient visit in medical centers, regional hospitals, district hospitals and local clinics, respectively. $W_{t}$ is monthly regular earnings, and $\zeta_{t}$ is the residuals. It is important to note that copayments per outpatient visit include the copayments for outpatient care services and prescription drugs [6]. Prior research on the influence of age structural transitions on policy responses utilizes time-varying impulse response functions to measure the responses of policy adjustments to variables of interest [35,36]. Following this line of research, we also applied the time-varying parameter vector autoregressive model (developed by Nakajima [8]) to estimate the time-varying impulse response functions that describe the response of medical center outpatient visits to upward adjustments in copayments. Thus, the time-varying impulse responses of medical center outpatient visits to one standardized unit shock in copayment, across different types of hospitals and local clinics (generated by the time-varying impulse response functions), were used to measure copayment policy effectiveness. For the sake of brevity, we will skip the technical details of the specification and estimation process of the time-varying parameter vector autoregressive model, and refer any interested reader to the online Supplementary Materials. Several model specification tests, such as the Hansen instability *Lc*, *Exp-F*, *Ave-F* and *Sup-F* tests, were employed to test for the null hypothesis of parameter stability within the vector autoregressive system for our time series data used in this study (see Chen [35] for details). 

### 2.2. Effect of Age Structural Transitions on Copayment Responses 

Since the effect of age structural transitions on policy effectiveness has been explained by previous studies [35,36], we specify the nonlinear relationship between age distribution and copayment policy effectiveness as follows:

(2) $$imq_{it}^{g}=\alpha_{0}^{g}+\sum_{w=1}^{W}\phi_{w}^{g}p_{wt}+\alpha_{1}^{g}cv_{t}+\xi_{t}^{g}\;\mathrm{for}\;g=m,\;a,$$

where $imq_{it}^{g}$ (*g* = *m* or *a*) was generated from the time-varying impulse response functions by estimating the time-varying parameter vector autoregressive model. It represents the effect of a change in copayment per medical center outpatient visit to various types of healthcare providers on medical center outpatient visits at time *t*, and its impacting time scale *i* (1, 2, 3, …, 12 months). $imq_{it}^{m}$ denotes the maximum (based on the minimal negative principle) response of medical center outpatient visits to one standardized unit change in the copayment per medical center outpatient visit, over the succeeding 12 months. Conventional microeconomic theory predicts that, other things being equal, an increase in copayment per medical center outpatient visit should decrease the number of medical center outpatient visits. Thus, negative values of $imq_{it}^{m}$ suggest that the copayment policy is effective in terms of an increase in the copayment per medical center outpatient visit.

Previous studies (such as Chen, Chi and Lin [3], and Chen, Liang and Lin [4], which modeled the discrete choice demand for outpatient care under Taiwan’s NHI system) suggested that regional hospitals and medical centers could be classified as one group of healthcare providers, due to the minor differences in the first contact user fees between these two providers. Following this line of classification, regional hospital outpatient care could be substituted for medical center outpatient care. In addition, one may expect that the outpatient care provided by regional hospitals, district hospitals and local clinics is complementary to that provided by medical centers, because local clinics (emphasizing primary care), district hospitals (dealing with secondary care), regional hospitals (responsible for tertiary care) and medical centers (treating the most complex diseases) were designed to constitute a (noncompulsory) referral system under Taiwan’s NHI system. In order to examine the possible relationships between outpatient care provided by different healthcare providers, we considered the accumulative maximum responses, of medical center outpatient visits (symbolized by $imq_{it}^{a}$ ) to the simultaneous change in copayment per visit by one standardized unit, from four providers (namely, medical centers, regional hospitals, district hospitals and local clinics). This procedure incorporates the same definition of copayment policy effectiveness as was used in generating the maximum response of medical center outpatient visits to the change in the copayment per medical center outpatient visit over the succeeding 12 months ( $imq_{it}^{m}$ ), with negative values of $imq_{it}^{a}$ indicating copayment policy effectiveness, with regard to a simultaneous increase in the copayment per outpatient visit from all providers. 

Furthermore, the proportion of the population in each individual age group *w* at time *t* (*t = 1, 2, 3 …, T*) was denoted by $p_{wt}$ (*w = 1, 2, 3, …, W*), and $cv_{t}$ indicates the control variables. $\alpha_{0}^{g}$, $\phi_{w}^{g}$, and $\alpha_{1}^{g}$ are the parameters corresponding to the constant term, the proportion of the population in age group *w*, and control variables, respectively. $\xi_{t}^{g}$ represents residuals. It is important to note that the model specification in Equation (2) includes proportions of the population from all age groups ( $p_{wt}$, *w = 1, 2, …, W*), so we were unable to estimate our empirical model due to the perfect collinearity problem of age distribution. To avoid this problem, we imposed some parametric restrictions on the $\phi_{w}^{g}$ parameters in Equation (2), based on Fair and Dominquez’s method, to estimate coefficients of $\phi_{w}^{g}$ (*w = 1, 2, 3, …, W*) [37]. In this way, we could apply the delta method to obtain the standard errors of $\phi_{w}^{g}$ (*w = 1, 2, 3, …, W*), and 90% confidence intervals for the estimated coefficients of $\phi_{w}^{g}$ (*w = 1, 2, 3, …, W*) could be established accordingly. Those estimated coefficients enabled us to portray the effects of age structural transitions on copayment policy responses.

The validation of statistical inferences generated from Equation (2) relies on the stationarity of time series data. In this study, we utilized the newly developed Fourier unit root test proposed by Chang, Lee and Chou [38]. The Fourier unit root test has been proven to perform better than conventional unit root tests, such as the Dickey‒Fuller test (a special case of the Fourier unit root test), in terms of the size and power properties of test statistics [38,39]. The results generated from the Fourier unit root tests suggest that most of our time series data support a rejection of the null hypothesis of the conventional Dickey‒Fuller unit root specification, in favor of the alternative hypothesis of the Fourier specification, and the stationarity of all variables used in this study is confirmed. For the sake of brevity, we once again skip the technical details of the model specifications of Fair and Dominquez’s method [37] and the Fourier unit root test [38,39], as well as their estimated empirical results. We refer any interested reader to the online Supplementary Materials.

### 2.3. Data and Variables 

Data for this research came from Taiwan’s National Health Insurance Research Database (NHIRD) [40], the Demographic Statistics Database (DSD) [41], and the Macroeconomics Statistics Database (MSD) [42], administered by the Taiwanese government. This study uses secondary data (i.e., monthly aggregate healthcare utilization data for all residents in Taiwan), and did not involve any human participants and/or tissue. The data collection process was approved by the Research Ethics Committee of Taichung Tzu Chi Hospital, with the Certificate of Exempt Review ID: REC106-28. Prior to modeling the responses of medical center outpatient visits to changes in copayment per outpatient visit for various types of healthcare providers, we defined and calculated the variables used in this study as follows: First, monthly outpatient visits per capita were computed as the monthly total outpatient visits to medical centers divided by the monthly total population. Second, copayments per outpatient visit to various providers (medical centers, regional hospitals, district hospitals and local clinics) were calculated as the monthly total copayment divided by the monthly total outpatient visits. Third, data for total outpatient visits and total copayments to various providers were retrieved from the NHIRD. Fourth, monthly regular earnings and total population per month were obtained from the MSD and DSD, respectively. Fifth, all the price variables were transformed into real price variables, at the 2011 price level, using the appropriate price indices (such as medical price index and labor wage index), and monthly medical center outpatient visits per capita were also transformed into annual outpatient visits by multiplying by 12. 

The responses of medical center outpatient visits to changes in copayment per outpatient visit for various types of healthcare providers, obtained by the time-varying parameter vector autoregressive model, were utilized to generate two measures of copayment policy effectiveness. The first is the maximum response (based on the minimal negative principle) of medical center outpatient visits over the 12 months to a change, by one standardized unit shock, in copayment per medical center outpatient visit. The other is the accumulative maximum response of medical center outpatient visits per capita to the simultaneous increase in copayment per outpatient visit, by one standardized unit of copayment, from all providers (i.e., medical centers, regional hospitals, district hospitals and local clinics). These unique measures were first generated using a total of 216 monthly observations over the period of January 1998‒December 2015. They were then transformed into quarterly data (by average, resulting in a total of 72 items of quarterly data, from the first quarter of 1998 to the fourth quarter of 2015) to serve as the dependent variable in the multiple linear regression model, while matching the quarterly frequency of some control variables used in the multiple linear regression model. The independent variables used in the multiple linear regression model are quarterly age distribution data, which include proportions of the population in seven age-specific groups (i.e., under 15, aged 15‒24, aged 25‒34, aged 35‒44, aged 45‒54, aged 55‒64 and 65 or over). These data were obtained from the DSD and MSD. Control variables, such as the contributions of the healthcare and social service sector to economic growth (measuring the prosperity of the healthcare industry), the unemployment rate (measuring business cycles), and the female labor participation rate (measuring socioeconomic transitions), were all retrieved from the MSD. 

## 3. Results

### 3.1. Time-Varying Parameter Vector Autoregressive Model

The upper part of Table 1 shows that the mean of medical center outpatient visits per capita was about 1.143 visits, and the average copayments per outpatient visit (at the 2011 price level) to medical centers, regional hospitals, district hospitals and local clinics were approximately NT$ 189, NT$ 157, NT$ 95 and NT$ 58, respectively. Monthly regular earnings at the 2011 price level are about NT$ 37,463. In addition, four parameter stability tests for the null hypothesis of the time-invariant parameter vector autoregressive model against the time-varying parameter vector autoregressive model are shown in the lower part of Table 1. The *Sup-F*, *Ave-F*, *Ave-F* and Hansen instability *L_c_* statistics [43,44] generated *p* values lower than the 5% significance level in all six equations within the vector autoregressive system. Therefore, the null hypothesis of parameter stability in the vector autoregressive system was soundly rejected, and these results validated the use of the time-varying parameter vector autoregressive model to evaluate the responses of medical center outpatient visits to the changes in copayment per outpatient visit for various types of healthcare providers.

**Figure 1:**
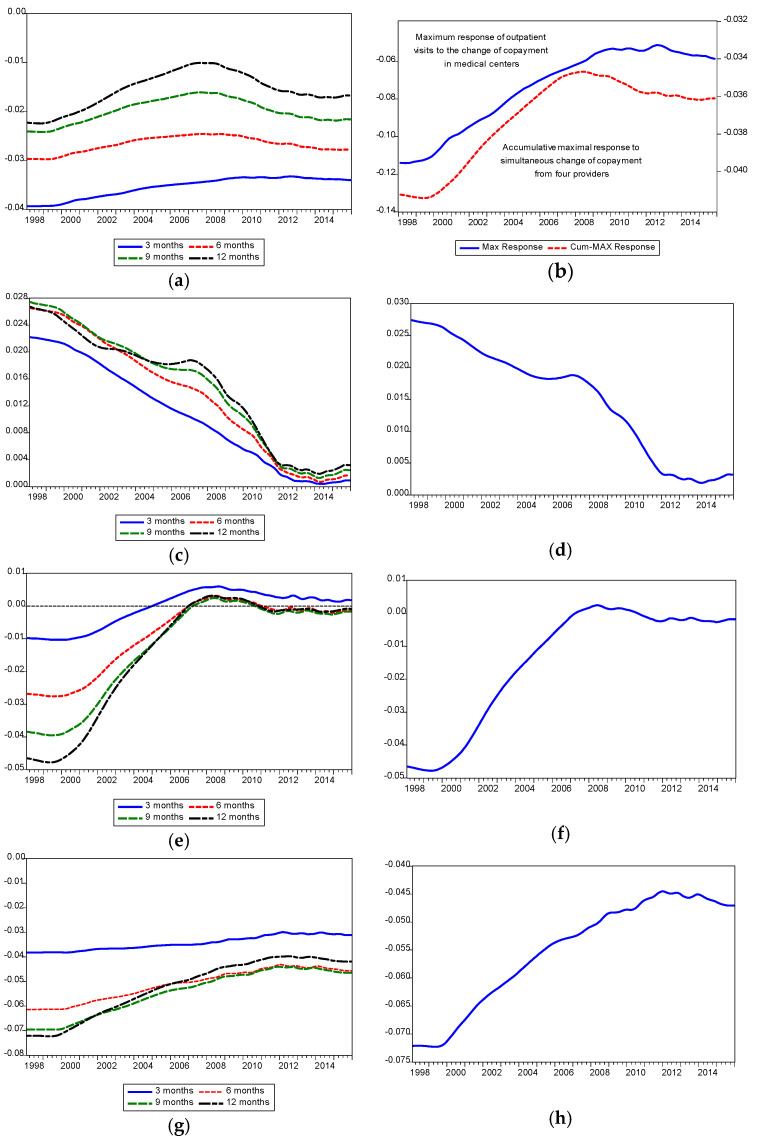
Impulse responses of medical center outpatient visits to copayment policy. (**a**) Response of medical center outpatient visits to 1 standardized unit change of copayment for medical centers. (**b**) Accumulative maximum response of medical center outpatient visits to 1 standardized unit change of copayment for medical centers (all providers). (**c**) Response of medical center outpatient visits to 1 standardized unit change of copayment for regional hospitals. (**d**) Maximum response of medical center outpatient visits to 1 standardized unit change of copayment for regional hospitals. (**e**) Response of outpatient visits to 1 standardized unit change of copayment for district hospitals. (**f**) Maximum response of outpatient visits to 1 standardized unit change of copayment for district hospitals. (**g**) Response of outpatient visits to 1 standardized unit change of copayment for clinics. (**h**) Maximum response of outpatient visits to 1 standardized unit change of copayment for clinics.

The propagation mechanisms of copayment impact, over the time scale of 1‒12 months during the period from January 1998 to December 2015, are displayed in Figure 1. The impulse responses of medical center outpatient visits to a positive shock of copayment per medical center outpatient visit, for the 3- to 12-month time scale, were all negative during our study period (see Figure 1a). These findings result in negative values for the maximum (based on the minimal negative principle) responses of medical center outpatient visits, over the 12 months following a positive shock to copayment per medical center outpatient visit (see Figure 1b). These results imply a negative price elasticity of demand for outpatient care in medical centers. Contrarily, the impulse responses of medical center outpatient visits to a positive shock of copayment per regional hospital outpatient visit, for the 3- to 12-month time scale, were all positive during the period from January 1998 to December 2015 (see Figure 1c). These findings show positive values in maximum (based on the maximal positive principle) responses of medical center outpatient visits, over the 12 months following a positive shock to copayment per regional hospital outpatient visit (see Figure 1d). These results indicate a positive cross-elasticity between medical center outpatient care and regional hospital outpatient care, and hence, the outpatient care provided by these two providers is interchangeable. 

As indicated in Figure 1e, the impulse responses of medical center outpatient visits to a positive shock of copayment per district hospital outpatient visit tend to be negative, for the 3- to 12-month time scale, during our study period. Therefore, we used the minimal negative principle to define maximum responses of medical center outpatient visits, over the 12 months following a positive shock to copayment per district hospital outpatient visit. It follows that maximum responses of medical center outpatient visits, over the 12 months following a positive shock, to copayment per district hospital outpatient visit were negative during most of our study period (see Figure 1f). Moreover, the impulse responses of medical center outpatient visits to a positive shock of copayment per local clinic outpatient visit, from the 3- to 12-month time scale, were all negative during our observed period (see Figure 1g). These findings show negative values in maximum (based on the minimal negative principle) responses of medical center outpatient visits, over the 12 months following a positive shock, to copayment per local clinic outpatient visit (see Figure 1h). These results suggest a negative cross-elasticity between medical center outpatient care and local clinic outpatient care, and therefore, the outpatient care provided by these two providers is likely to be complementary. The relationship between medical center outpatient visits and copayment per outpatient visit, for the four providers, was summarized as the accumulative maximum response of medical center outpatient visits to a simultaneous increase in copayment per visit by one standardized unit, for medical centers, regional hospitals, district hospitals and local clinics (see the red dotted line in Figure 1b). The negative values in the accumulative maximum response of medical center outpatient visits to copayment adjustments reveal that patients were responsive, and medical center outpatient visits reduced as the prices of outpatient care per visit were adjusted upwards under Taiwan’s NHI system.

### 3.2. Effect of Age Structural Transitions on Copayment Responses

**Figure 2:**
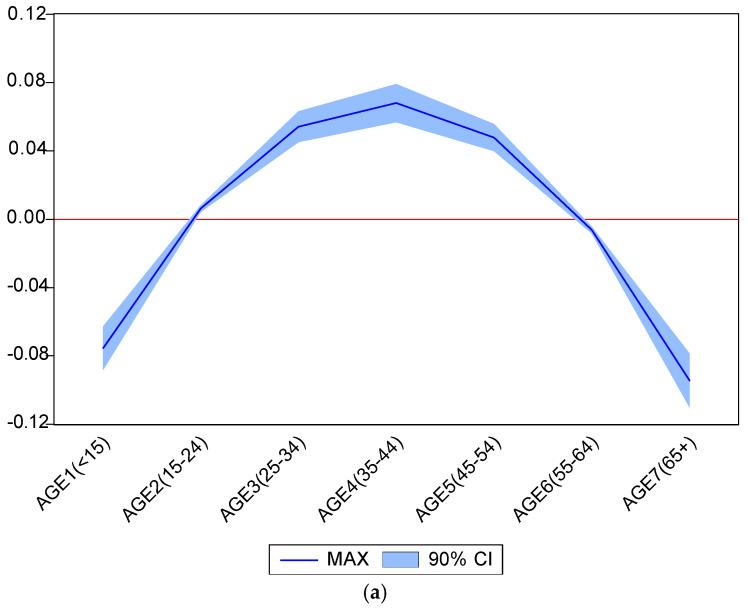

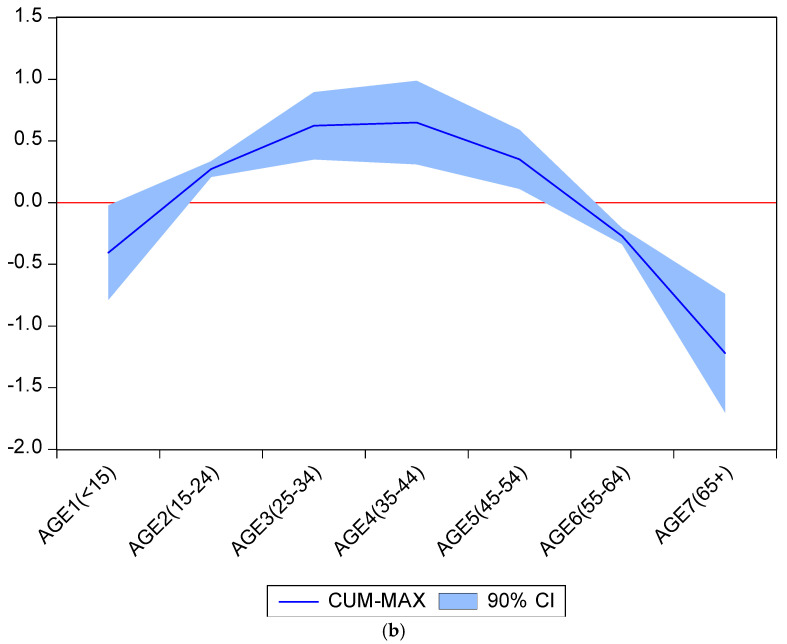
The effect of age distribution on copayment policy responses. (**a**) Effect of age distribution on the maximal responses of medical center outpatient visits to the change in copayment from medical centers. (**b**) Effect of age distribution on the accumulative maximal response of medical center outpatient visits to simultaneous change of copayment from all providers.

As shown in Table 2, the average maximum impulse responses of medical center outpatient visits to one standardized unit change of copayment per outpatient visit, for medical centers, regional hospitals, district hospitals and local clinics, were −0.036, 0.015, −0.015 and −0.055, respectively, and the means of accumulative maximum impulse responses of medical center outpatient visits to simultaneously increasing copayments per outpatient visit, by one standardized unit, for the four providers was −0.090, during the period from the first quarter of 1998 to the fourth quarter of 2015. These results are accordant with the interrelationship between medical center outpatient visits and copayments per outpatient visit from the four providers, illustrated in Figure 2. The average proportions of the population in seven age-specific groups during our observed period ranged from 9.9% (aged 65 and above) to 19.9% (age < 15). The average contribution of the health and social services sector to economic growth was about 8.3% over the period from the first quarter of 1998 to the fourth quarter of 2015. This result shows that Taiwan’s healthcare industry was prosperous during our study period, considering the average 4.07% economic growth rate during the same period [45]. The means of unemployment rate and female labor participation rate were approximately 4.19% and 48.48%, respectively. The former result (unemployment rate less than 5%) shows that Taiwan was in full employment status, and the latter result indicates that females played an important role in productivity during our study period. 

The empirical results for the multiple linear regression model are presented in Table 3. It is important to note that the negative values of the (accumulative) maximum responses of medical center outpatient care visits to the (simultaneous) change of copayment per outpatient visit for providers (medical centers, regional hospitals, district hospitals and local clinics) indicate copayment policy effectiveness. As shown in Table 3, the proportions of the population in seven age-specific groups have statistically significant impacts on copayment policy effectiveness. Figure 2a further illustrates the inverse U shape of the effect of age distribution on maximum responses of medical center outpatient care visits to the change of copayment per medical center outpatient visit. Figure 2b demonstrates the same shape for the effect of age distribution on accumulative maximum responses of medical center outpatient care visits to the simultaneous change of copayment per outpatient visit for all providers (namely, medical centers, regional hospitals, district hospitals and local clinics). Specifically, the proportions of the population in four major working age groups (i.e., aged 15‒24, aged 25‒34, aged 35–44 and aged 45‒54 groups) are positively correlated with these two measures of copayment policy effectiveness, but the proportions of the population in the two older age groups [the elderly (age ≥ 65) and aged 55‒64] and the children group (age < 15) have negative effects on the accumulative maximum and maximum responses of medical center outpatient visits to copayment policy change. In addition, the prosperity of the healthcare and social services industries could reinforce copayment policy effectiveness, since the estimated coefficients of the contribution of the healthcare and social services sector to economic growth is significantly negative. The estimated coefficients of the female labor participation rate are significantly positively at the 1% significance level, indicating that female participation in productivity would mitigate the effectiveness of copayment policy, possibly due to increased income resources coming into the household. 

## 4. Discussion 

Since the proportions of the population making up the bulk of the workforce (ages 15 to 54) have significantly positive effects on the accumulative maximum and maximum responses of medical center outpatient visits to upward adjustments in copayment, one may expect that copayment policy would be less effective when these major working populations expand. However, the proportions of the population in the two older age groups [55–64 and the elderly (age ≥ 65)] and the children group (age < 15) generate significantly negative effects on these two measures of copayment policy effectiveness, implying that copayment policy would be more effective when the populations of children, and those aged 55 years or above, grow. These findings are different from previous studies investigating the impact of age structural transitions on monetary policy responses, wherein population ageing is seen to attenuate the effectiveness of monetary policy (i.e., the responses of economic growth or unemployment rates to the adjustment of interest rates) [35,36]. It is worth noting that copayments put a direct price on a very specific activity—obtaining outpatient care services from different providers—whereas the monetary policy (in terms of adjusting interest rates to accomplish some economic targets) is an increase in the price of borrowing, and has effects on a range of economic activities. Population ageing mitigates the effects of monetary policy because the elderly have fewer choices than the young. That is, the young consume, save, and invest in human capital. The elderly are likely to just consume. In the case of healthcare services, the elderly consume a lot more healthcare services, thereby making them more price-sensitive, which would discourage consumption. A recent study conducted by Nillsson and Paul showed the elastic demand for children’s and adolescents’ healthcare services; the response of cost-sharing to children’s and adolescents’ healthcare utilization is negatively associated with their parental income [19]. Note that the real wage in Taiwan continuously decreased during our study period. The real wage in 2015 was around the same level as in 1999 [46]. More severe family income constraints in recent years, due to economic fluctuation, may be one of the crucial factors that explain why copayment policy effectiveness is positively correlated with children (age < 15). In addition, there is more dependency with respect to decision-making in the elderly (age ≥ 65) and children (age < 15) age groups. One might presume that people making decisions (likely those aged 16‒54) are not necessarily “perfect agents”, and might be making health decisions that are not in the best interests of the dependents (age < 15, and age ≥ 65) they have charge over. This might be another reason why children (age < 15) and the two older age groups [aged 55‒64 and the elderly (age ≥ 65)] are more sensitive to copayment adjustments than their counterparts. In addition, despite increases in copayment fees for outpatient visits having a lower impact on working people (aged 15‒54), based on Figure 2a,b, a policy of reducing copayment fees is likely to increase health and long-term care expenditure for the elderly, and thus put a financial burden on the working groups (aged 15‒54). 

Furthermore, evidence from our time-varying impulse response plots in Figure 1 indicated that medical center outpatient care is complementary with the outpatient care provided by district hospitals and local clinics, but could be replaced by regional hospital outpatient care. If the relationships between medical center outpatient care and outpatient care provided by other healthcare providers (such as local clinics, regional hospitals and district hospitals) are taken into account, the proportions of the population in the two older age groups [aged 55‒64 and the elderly (age ≥ 65)] and children (age < 15) could be seen to have a much stronger effect on copayment policy effectiveness. This is because copayment adjustment in medical center outpatient care is most likely to raise the price of outpatient care provided by other healthcare providers, based on the expectations imposed on the economic agents. This is supported by the fact that the accumulative maximum responses of medical center outpatient visits to copayment policy change are much more significant than the maximum responses of medical center outpatient visits to copayment policy change, as we can see by noting that the scale of the vertical axis in Figure 2b is at least five times higher than that in Figure 2a. As a result, copayment policy would be even more effective in decreasing outpatient care utilization in medical centers, from the perspective of the dynamic interaction effect among different healthcare providers under Taiwan’s NHI system. 

The discussion in the previous paragraph should be of particular concern, because Taiwan is now experiencing rapid population ageing in terms of its historical longevity (life expectancy above 80) and low fertility (around one child per woman) [35]. Because of the ageing trend of the population, copayment policy will influence the elderly population the most in the near future. Consequently, an effective copayment policy is most likely to increase the financial burden of the elderly, and possibly force some elderly people, particularly the poor and the sick, who have an urgent need for healthcare services, to seek alternative treatment (or self-treatment) rather than the orthodox healthcare services provided by Taiwan’s NHI system [3,4,7]. It follows that fundamental equality of access to healthcare services under Taiwan’s NHI would gradually diminish as the elderly population rises. Therefore, policymakers should be warned against a possible pricing-out effect (meaning that the price of healthcare services is high enough to create a barrier preventing disadvantaged groups, such as the elderly, from accessing healthcare services) due to copayment policy change. A strategic intervention to combat the adverse effects of an increase in copayment fees could involve subsidizing healthcare services for elderly people with chronic diseases or low income. 

This study provides two innovative contributions, beyond those of prior studies on the effect, of copayment on healthcare utilization. First, we adopted the time-varying parameter vector autoregressive model developed by Nakajima [8], coupled with the multiple linear regression model, to establish a link between age distribution and copayment policy effectiveness (in terms of the negative response of medical center outpatient visits to upward copayment adjustment) for the first time. Second, the stationarity of time series variables used in this study was proved by the newly developed Fourier unit root test, which has better power and size properties than conventional unit root tests [38,39]. Third, our multiple linear regression model includes the whole range of age distribution, rather than a single measure of population ageing, so it can assess the nonlinear effect of age structural transitions on copayment policy responses. 

Nevertheless, a limitation inherent in this study is the time series methodology used for our investigation into the effect of age structural transitions on policy effectiveness. We are aware that the age distribution and medical center outpatient care utilization data used for our analyses belong to aggregated data, and the empirical results obtained from our empirical models should not refer to individual behavioral change, exhibited at one’s specific age in response to copayment adjustments on medical center outpatient visits, in order to avoid the ecological fallacy. Additionally, we did not apply other time series methodologies (such as the interrupted time series analyses for multiple groups) due to the lack of outpatient care utilization by multiple groups. Finally, the statistical inferences obtained from our time series analyses are confined to the long-term impact of age structural transitions on copayment policy effectiveness, because the change in age distribution is most likely to be a long-term process. 

## 5. Conclusions

Copayment, referring to the specified amount that the patient has to pay for each healthcare service received, has served as a means by which to limit healthcare utilization under Taiwan’s NHI system [6,7]. Previous studies of the relationship between copayment adjustment and healthcare utilization suggest that copayment adjustments have the positive effect of controlling healthcare utilization under Taiwan’s NHI system [7,31,32,33,34]. Nevertheless, Taiwanese population ageing is notoriously fast. It will take only 33 years for Taiwan’s demographic to transition from an ageing to a hyper-aged society [9]. As the Taiwanese population ages, an understanding of the effect of age structural transitions on copayment policy will become important in assisting policymakers in preparing strategic interventions to decrease the adverse impact of population ageing on the healthcare system. Hence, this study enriches the health economic literature, focusing on the impact of an ageing population on the healthcare system by demonstrating the time-varying impulse responses of medical center outpatient visits to copayment policy adjustments, and the impact of age structural transitions on copayment policy effectiveness. 

Specifically, the results obtained from our empirical models suggest that copayment policy effectiveness (in terms of the negative response of medical center outpatient visits to upward copayment adjustment) is positively correlated with the proportions of the population in the two older age groups [aged 55‒64 and the elderly (age ≥ 65)] and the children group (age < 15), but is negatively correlated with the proportion of the population aged 15‒54. The tendency of age distribution to affect the responses of medical center outpatient visits to copayment policy adjustments suggests that a copayment policy could be more effective in an ageing society. Therefore, policymakers should be concerned about the adverse effects copayment adjustments will have on the elderly population, such as deteriorating health, increasing financial burdens, and pricing some elderly patients out of Taiwan’s NHI system. One of the strategic schemes to limit these adverse effects of copayment adjustments is to provide a subsidy of healthcare services for the poor and those in greater need of healthcare services. We encourage future research into a tiered copayment system, and into projecting the extent of the pricing-out effect in relation to Taiwanese age structural transitions. 

## Figures and Tables

**Table 1:** Parameter stability tests for the time-varying parameter vector autoregressive model.

Descriptive Statistics ^†^										
Variables	Description				Mean		SD	Min		Max
OVC	Outpatient visits per capita in medical centers (transformed to annual visits by multiplying by 12)				1.143		0.355	0.470		1.709
CPM	Copayment per medical center outpatient visit at 2011 price level (NT$)				188.768		21.933	158.745		251.809
CPR	Copayment per regional hospital outpatient visit at 2011 price level (NT$)				157.015		20.077	125.248		217.197
CPD	Copayment per district hospital outpatient visit at 2011 price level (NT$)				94.736		22.449	69.935		186.882
CPC	Copayment per clinics outpatient visit at 2011 price level (NT$)				58.475		10.937	46.890		128.503
INC	Monthly regular earnings at 2011 price level (NT$ 1000)				37.463		0.989	35.620		39.633
**Parameter Stability Tests for VAR**(**1**) **System**^‡^										
Stability Tests	OVC Equation	CPM Equation	CPR Equation	CPD Equation		CPC Equation			INC Equation	
	Statistics (*p*-value)	Statistics (*p*-value)	Statistics (*p*-value)	Statistics (*p*-value)		Statistics (*p*-value)			Statistics (*p*-value)	
*Sup-F*	17.192 (0.00)	7.421 (0.00)	28.334 (0.00)	8.633 (0.00)		14.979 (0.00)			5.549 (0.00)	
*Ave-F*	10.075 (0.00)	1.290 (0.02)	9.425 (0.00)	1.403 (0.04)		3.457 (0.00)			3.748 (0.00)	
*Exp-F*	6.591 (0.00)	1.252 (0.21)	4.345 (0.00)	1.115 (0.13)		4.341 (0.00)			1.975 (0.00)	
*L_c_*	2.441 (0.00)	7.614 (0.00)	8.924 (0.00)	4.899 (0.00)		23.934 (0.00)			2.395 (0.00)	

^†^ Note: 1 USD = 30 NT$. The whole sample period spanned from January 1998 to December 2015, generating a total of 216 monthly observations. ^‡^ Standardized variables were used to estimate the time-varying parameter vector autoregressive (TVP-VAR) model. One lag was selected by the convergence of TVP-VAR model; VAR is the abbreviation for “vector autoregressive”, and VAR(1) means the VAR model with one lag period. The *p* values for the *Sup-F Ave-F* and *Exp-F* tests were calculated based on Hansen [44]. The *p* values for *L_c_* were calculated based on Hansen [43].

**Table 2:** Descriptive statistics for the multiple linear regression model ^†^.

Variables	Description	Mean	SD	Min	Max
MRM	Maximal (based on minimal negative principle) response of medical center outpatient visits per capita to a standardized unit change of the copayment per medical center outpatient visit within a 12-month period.	−0.036	0.002	−0.040	−0.033
MRR	Maximal (based on maximal positive principles) response of medical center outpatient visits per capita to a standardized unit change of the copayment per regional hospital outpatient visit within a 12-month period.	0.015	0.009	0.002	0.027
MRD	Maximal (based on minimal negative principle) response of medical center outpatient visits per capita to a standardized unit change of the copayment per district hospital outpatient visit within a 12-month period.	−0.015	0.018	−0.048	0.002
MRC	Maximal (based on minimal negative principle) response of medical center outpatient visits per capita to a standardized unit change of the copayment per clinic outpatient visit within a 12-month period.	−0.055	0.009	−0.072	−0.045
Cum-Max	Accumulative maximal response of medical center outpatient visits per capita to a simultaneous increase in copayment per outpatient visit by a standardized unit for medical centers, regional hospitals, district hospitals and local clinics. Namely, Cum-Max = MRM + MRR + MRD + MRC.	−0.090	0.023	−0.133	−0.065
Age 1	Proportion of the population in the children (age < 15) group	0.199	0.030	0.153	0.249
Age 2	Proportion of the population in the aged 15‒24 group	0.142	0.012	0.127	0.161
Age 3	Proportion of the population in the aged 25‒34 group	0.160	0.006	0.144	0.169
Age 4	Proportion of the population in the aged 35‒44 group	0.162	0.005	0.155	0.168
Age 5	Proportion of the population in the aged 45‒54 group	0.143	0.017	0.103	0.158
Age 6	Proportion of the population in the aged 55‒64 group	0.094	0.023	0.071	0.137
Age 7	Proportion of the population in the elderly (age ≥ 65) group	0.099	0.012	0.080	0.124
CRH	Contribution of the healthcare and social services sector to economic growth	0.083	0.087	−0.160	0.320
UR	Unemployment rate (%)	4.192	0.829	2.453	6.080
FLPR	Female labor participation rate (%)	48.476	1.807	45.250	50.887

^†^ The whole sample period spanned from the first quarter of 1998 to the fourth quarter of 2015, generating a total of 72 quarterly observations.

**Table 3:** Effects of age structural transitions on copayment policy effectiveness.

Age Distribution	MRM ^†^		Cum-Max ^†^	
	Coefficient	*T*-value ^†^	Coefficient	*T*-Value ^†^
AGE1 (<15)	−0.075	−9.874 ***	−0.405	−1.745 *
AGE2 (15‒24)	0.006	5.457 ***	0.272	7.172 ***
AGE3 (25‒34)	0.054	10.016 ***	0.624	3.779 ***
AGE4 (35‒44)	0.068	10.058 ***	0.650	3.162 ***
AGE5 (45‒54)	0.048	10.007 ***	0.352	2.424 **
AGE6 (55‒64)	−0.006	−5.457 ***	−0.272	−7.172 ***
AGE7 (>64)	−0.094	−9.937 ***	−1.220	−4.193 ***
Control Variables	Coefficient	*T*-value ^‡^	Coefficient	*T*-value ^‡^
CRH	−0.001	−1.694 *	−0.017	−1.915 *
Ln (UR)×10^−2^	0.024	0.913	0.736	1.163
Ln (FLPR)	0.020	5.374 ***	0.772	6.472 ***
Constant	−0.118	−7.890 ***	−3.160	−6.791 ***

^†^ MRM represents the maximal (based on minimal negative principle) response of medical center outpatient visits per capita to a standardized unit change of the copayment per medical center outpatient visit within a 12-month period. Cum-Max denotes the accumulative maximal response of medical center outpatient visits per capita to a simultaneous increase in copayment per outpatient visit by a standardized unit for medical centers, regional hospitals, district hospitals and local clinics. *, **, *** represent the 10%, 5% and 1% significance levels, respectively. *T*-values were estimated through the delta method. ‡ *T*-values were computed by dividing the estimated coefficients by the Newey‒West standard errors. CHR represents the contribution of the healthcare and social services sector to economic growth. UR and FLPR denote the unemployment rate and female labor participation rate, respectively.

## Supplementary Materials

The following are available online at https://www.mdpi.com/1660-4601/17/12/4183/s1. This section consists of model specifications for the time-varying parameter vector autoregressive (TVP-VAR) model with stochastic volatility, the parametric age response function and Fourier unit root tests, and results obtained from the Fourier unit root tests and the parametric age response function.
