# Supplementary material for: Age Structural Transitions and Copayment Policy Effectiveness: Evidence from Taiwan’s National Health Insurance System
Source: Int J Environ Res Public Health. 2020 Jun 12;17(12):4183. doi: 10.3390/ijerph17124183 (PMC7344636; doi:10.3390/ijerph17124183)
Supplement: Supplementary file 1 [file ijerph-17-04183-s001.pdf]

Appendix: Online supplementary materials for review purpose

1. TVP-VAR model with stochastic volatility

The aim of this study is to investigate the relationship between demographic structure and copayment policy effectiveness (particularly in medical centers) under Taiwan's NHI system. To understand the response of medical center outpatient visits to outpatient visit copayment adjustments for medical centers, regional hospitals, district hospitals, and local clinics, we specified the time-varying parameter vector autoregressive (TVP-VAR) model with stochastic volatility (introduced by Nakajima (2011)) as follows:

$$\mathbf{x_t} = \mathbf{c}_t + \mathbf{B}_{1t}\mathbf{x}_{t-1} + \mathbf{B}_{2t}\mathbf{x}_{t-2} +,....,\mathbf{B}_{st}\mathbf{x}_{t-s} + \mathbf{e}_t \quad \text{for } t=s+1,\, s+2,.....,n \qquad \text{(A1)}$$

where, $\mathbf{x}_t = [q_t^m, p_t^m, p_t^r, p_t^d, p_t^c, W_t]'$ is a vector including medical center outpatient visits per capita (q_t^m), monthly regular earnings (W_t), and copayments per outpatient visit in medical centers (p_t^m), regional hospitals (p_t^r), district hospitals (p_t^d), and local clinics (p_t^c). $\mathbf{B}_{1t}, \mathbf{B}_{2t},....,\mathbf{B}_{st}$ are 6×6 matrices of time-varying parameters. $\mathbf{e}_t \sim N(\mathbf{0}, \mathbf{\Omega}_t)$. Because the recursive identification was imposed in equation (A1), $\mathbf{\Omega}_t$ could be further decomposed into $\mathbf{\Omega}_t = \mathbf{A}_t^{-1}\mathbf{\Sigma}_t\mathbf{\Sigma}_t\mathbf{A}_t'^{-1}$ (which is a 6×6 time-varying covariance matrix). $\mathbf{A}_t$ is a lower-triangle matrix with the diagonal elements equaling one and $\mathbf{\Sigma}_t = diag(\sigma_{1t}, \sigma_{2t}, \sigma_{3t}, \sigma_{4t}, \sigma_{5t}, \sigma_{6t})$. Nakajima (2011) specified time-varying parameters based on the random walk process assumption, so the time-varying parameters in equation (A1) are given by

$$\begin{cases} \boldsymbol{\beta}_{t+1} = \boldsymbol{\beta}_t + \mathbf{u}_{\boldsymbol{\beta}t} \\ \mathbf{a}_{t+1} = \mathbf{a}_t + \mathbf{u}_{\mathbf{a}t} \\ \mathbf{h}_{t+1} = \mathbf{h}_t + \mathbf{u}_{\mathbf{h}t} \end{cases}, \quad \begin{pmatrix} \boldsymbol{\varepsilon}_t \\ \mathbf{u}_{\boldsymbol{\beta}t} \\ \mathbf{u}_{\mathbf{a}t} \\ \mathbf{u}_{\mathbf{h}t} \end{pmatrix} \sim N\left(\mathbf{0}, \begin{pmatrix} \mathbf{I} & \mathbf{0} & \mathbf{0} & \mathbf{0} \\ \mathbf{0} & \mathbf{\Sigma}_{\boldsymbol{\beta}} & \mathbf{0} & \mathbf{0} \\ \mathbf{0} & \mathbf{0} & \mathbf{\Sigma}_{\mathbf{a}} & \mathbf{0} \\ \mathbf{0} & \mathbf{0} & \mathbf{0} & \mathbf{\Sigma}_{\mathbf{h}} \end{pmatrix}\right) \quad \text{for } t=s+1,...,n \qquad \text{(A2)}$$

where, $\mathbf{h}_t = [\log\sigma_{1t}^2, \log\sigma_{2t}^2, \log\sigma_{3t}^2, \log\sigma_{4t}^2, \log\sigma_{5t}^2, \log\sigma_{6t}^2]$, and $\boldsymbol{\beta}_t$ and $\mathbf{a}_t$ represent the stacked row vector of $\mathbf{B}_{1t}, \mathbf{B}_{2t},....,\mathbf{B}_{st}$ and the stacked row vector of the free lower-triangle elements of $\mathbf{A}_t$, respectively. $\boldsymbol{\beta}_{s+1} \sim N(\mathbf{m}_{\boldsymbol{\beta}0}, \mathbf{\Sigma}_{\boldsymbol{\beta}0})$, $\mathbf{a}_{s+1} \sim N(\mathbf{m}_{\mathbf{a}0}, \mathbf{\Sigma}_{\mathbf{a}0})$, and $\mathbf{h}_{s+1} \sim N(\mathbf{m}_{\mathbf{h}0}, \mathbf{\Sigma}_{\mathbf{h}0})$. $\mathbf{\Sigma}_{\mathbf{a}}$ and $\mathbf{\Sigma}_{\mathbf{h}}$ are diagonal. $\boldsymbol{\varepsilon}_t = \mathbf{\Sigma}_t^{-1}\mathbf{A}_t\mathbf{e}_t$. The impulse-responses of the effect of one standardized unit shock in copayment ($\partial\mathbf{x_{t+1}}/\partial\boldsymbol{\varepsilon}_\mathbf{t}$) could be estimated from the Cholesky decomposition of $\mathbf{A}_t^{-1}\mathbf{\Sigma}_t$. The Bayesian Markov Chain Monto Carlo (MCMC) method was adopted to estimate the TVP-VAR model with stochastic volatility described in equations (A1)-(A2). Lags in the TVP-VAR system were chosen to be one based on the grid search for convergence of the Bayesian MCMC method, and therefore, 21 parameters (15 parameters for $\mathbf{A}_t$ and 6 parameters for $\mathbf{h}_t$) were estimated to generate the impulse-responses of the effect in copayment adjustment ($\partial\mathbf{x_{t+1}}/\partial\boldsymbol{\varepsilon}_\mathbf{t}$) through the Cholesky decomposition of $\mathbf{A}_t^{-1}\mathbf{\Sigma}_t$. The Bayesian MCMC estimation of the TVP-VAR model with stochastic volatility was based on 20,000 runs of the Gibbs Sampler, and the first 2,000 draws were discarded for the purpose of convergence to ergodic distribution. There are two advantages of the Bayesian MCMC estimation used here. First, it partially solves the limited sample size (216 monthly data points used in this study), leading to lack of the power of inference by the simulation method. Second, the time-varying estimated parameters in $\mathbf{A}_t^{-1}\mathbf{\Sigma}_t$ were generated by

the random walk process imposed on equation (A2) that largely reduced the estimated parameters in the TVP-VAR model with stochastic volatility.

2. Model specification for the parametric age response function

Following the parametric age response function proposed by Fair and Dominquez (1991), the nonlinear relationship between age distribution and copayment policy effectiveness can be written by

$$imq_{it}^{g} = \alpha_0^{g} + \sum_{j=1}^{J} \varphi_j^{g} p_{jt} + \alpha_1^{g} cv_t + \xi_t^{g} \quad \text{for } g=m,\, a \qquad \text{(A3)}$$

where, imq_{it}^{g} (*g*=*m* or *a*) represents the effect of copayment per medical center outpatient visit change from various types of healthcare providers on medical center outpatient visits at time *t* and its impacting timescale *i* (1,2,3,…,12 months). imq_{it}^{m} denotes the maximum (based on the minimal negative principle) response of medical center outpatient visits to one standardized unit change of the copayment per medical center outpatient visit over the subsequent 12 months. imq_{it}^{a} indicates the accumulative maximum response of medical center outpatient visits to the simultaneous change in copayment per visit by one standardized unit from four providers (namely, medical centers, regional hospitals, district hospitals, and local clinics).

The share of total population in age group *j* at time *t* was denoted by p_{jt} (*j=1,2,...,J*), and cv_t indicates the control variables. α_0^{g} , φ_j^{g} , and α_1^{g} are the parameters corresponding to the constant term, age group *j*, and control variables, respectively. ξ_t^{g} represents residuals. Note that the model specification in equation (A3) includes all age groups (p_{jt} , *j=1,2,...,J*), which creates a perfect collinearity in the estimation process. To avoid this collinearity problem, Fair and Dominquez (1991) proposed the imposition of a restriction on the φ_j^{g} parameters as follows:

$$\varphi_j^{g} = \delta_0 + \delta_1 j + \delta_2 j^2 \quad \text{and} \quad \sum_{j=1}^{J} \varphi_j^{g} = 0 \qquad \text{(A4)}$$

If we substitute equation (A4) into equation (A3), we obtain our final model specification for the multiple linear regression model as follows:

$$imq_{it}^{m} = \alpha_0^{g} + \delta_1 z_{1t} + \delta_2 z_{2t} + \alpha_1^{g} cv_t + \xi_t^{g} \quad \text{and} \quad \begin{cases} z_{1t} = \sum_{j=1}^{J} j p_{jt} - J^{-1} \sum_{j=1}^{J} j \sum_{j=1}^{J} p_{jt} \\ z_{2t} = \sum_{j=1}^{J} j^2 p_{jt} - J^{-1} \sum_{j=1}^{J} j^2 \sum_{j=1}^{J} p_{jt} \end{cases} \qquad \text{(A5)}$$

where, z_{1t} and z_{2t} are two transformed variables of age distribution based on Fair and Dominquez (1991), and other notations used in equation (A5) are the same as those used in equation (A3). δ_1 and δ_2 are parameters corresponding to z_{1t} and z_{2t} , respectively. The possible nonlinear effect of age distribution on copayment policy effectiveness can be established by the estimated coefficients of φ_j^{g} (*j=1,2,...,J*) through the parametric restrictions imposed on the φ_j^{g} parameters in equation (A3) and the estimated coefficients of δ_1 and δ_2 .

3. Fourier unit root test

The validation of statistical inferences generated from equation (A3) relies on the stationarity of time series data. In this study, we utilized the newly developed Fourier unit root test proposed by Chang, Lee and Chou (2012). The Fourier unit root test has been proved to perform better than conventional unit root tests, such as the Dickey-Fuller test, in terms of the size and power properties of test statistics (Chang, Lee and Chou, 2012; Enders and Lee, 2012). Specifically, the data-generating process (DGP) for the Fourier unit root test can be given by

$$y_t = \theta_0 + \theta_1 t + \lambda_1 \sin(2\pi kt/T) + \lambda_2 \cos(2\pi kt/T) + \upsilon_t, \upsilon_t = \rho\upsilon_{t-1} + \eta_t, t=1,2,3,\ldots,T \qquad \text{(A6)}$$

where, θ_0 and θ_1 are the parameters associated with constant and time trend, respectively. k denotes the optimal frequency selected for the approximation of smooth transition. λ_1 and λ_2 measure the amplitude and displacement of the frequency component, respectively. The error term (υ_t) has the autoregressive form (ρ) with one lag, and η_t is white noise. It is important to address that the DGP for the Dickey-Fuller unit root tests is a special case by setting $\lambda_1 = \lambda_2 = 0$ in equation (A6). In contrast to the conventional Dickey-Fuller unit root specification, equation (A6) can be rewritten as the following expression

$$\Delta y_t = \gamma\tilde{S}_{t-1} + \phi_0 + \phi_1\Delta\sin(2\pi kt/T) + \phi_2\Delta\cos(2\pi kt/T) + \mu_t, \qquad \text{(A7)}$$

where, Δ is the difference operator, $\tilde{S}_{t-1}$ is the de-trended series. ϕ_j (j=0,1,2) and γ are estimated coefficients. μ_t is assumed to satisfy the serial correlation and heterogeneity conditions indicated in Phillips and Perron (1988). Equation (A7) can be augmented with the lag values of $\Delta\tilde{S}_{t-j}$, j=1,2,…,L in order to remove the remaining serial correlation. Chang, Lee and Chou (2012) proposed the *LM* statistic (denoted by τ_{LM}) to test for the null hypothesis of the unit root (γ=0) against the alternative hypothesis of stationarity. The *LM* statistic (τ_{LM}) depends only on the frequency *k*, but it is invariant to other parameters in the DGP (Chang, Lee and Chou, 2012; Enders and Lee, 2012). Therefore, the empirical procedure used to proceed with the Fourier unit root test consisted of three steps: First, we determined the frequency *k* (*k*<3 suggested by Enders and Lee (2012)) through minimizing the sum of squared residuals. Second, the *F*(*k*) test was used to test for the null hypothesis of the DGP for the Dickey-Fuller unit root test against the alternative hypothesis of the Fourier unit root test. As indicated in Enders and Lee (2012), asymptotic distribution of *F*(*k*) is non-standard, and the critical values of *F*(*k*) for 10%, 5%, and 1% levels of significance were obtained from Ender and Lee (2012). Third, the *LM* statistic (τ_{LM}) was estimated from equation (8). However, since its asymptotic distribution is also non-standard (Chang, Lee and Chou, 2012; Enders and Lee, 2012), the 10%, 5%, and 1% critical values of the *LM* statistic (τ_{LM}) were calculated using 20,000 bootstrapping replications.

4. Results for Fourier unit root tests

As indicated in equations (A2)-(A5), the shares of the total population in *J* age-specific groups, *j*=1(age<15), 2(15-24 years old), 3(25-34 years old)..,7(age ≥65), were as further transformed into two demographic measures, z1 (the linear transformation of the age distribution) and z2 (the quadratic transformation of the age distribution). The means of z1 and z2 are -0.413(with Standard Deviation=0.212), and -3.374(with Standard Deviation=1.588), respectively.

Table A1 Fourier Unit Root Tests†

Variables	$\hat{k}$	【Ho:ADF vs H1: Fourier ADF】 $F(\hat{k})$	Lags of $\Delta\tilde{S}_{t-j}$	【Ho: I(1) vs H1: I(0)】 $\tau_{LM}(\hat{k})$ [ADF]	Critical values 10%	5%	1%
Cum-Max	1	133.393***	3	-2.275**	-1.378	-1.757	-2.465
MRM	1	10.195**	4	-3.135 ***	-1.418	-1.797	-2.553
z1	1	8.682*	4	-10.126***	-1.307	-1.680	-2.450
z2	1	5.643	4	-10.267***	-1.308	-1.694	-2.429
	--	------	[2]	[-4.614***]	[-3.166]	[-3.476]	[-4.093]
CRH	1	47.008***	2	-1.664*	-1.458	-1.828	-2.534
ln(UR)	2	12.345***	4	-19.141***	-1.872	-2.242	-2.996
ln(FLPR)	1	32.158***	4	-7.251***	-1.553	-1.942	-2.660

† The critical values of $F(\hat{k})$ for 10%, 5%, and 1% levels of significance obtained from Ender and Lee (2012) were 7.78, 9.14, and 12.21, respectively. The critical values of the *LM* (*ADF*) statistics for 10%, 5%, and 1% levels of significance were obtained by the bootstrap method with 20,000 replications (MacKinnon's method, see MacKinnon(1996) for details).UR and FLPR denote unemployment rate, and female labor participation rate, respectively.

Table A1 presents the Fourier unit root tests for the variables used in the multiple linear regression model. The procedure proposed by Enders and Lee (2012) selects two as the optimal frequency ($\hat{k}$) for the unemployment rate, and one as the remaining variables used to identify the nonlinear effect of age distribution on copayment policy effectiveness. The $F(\hat{k})$ statistics shown in Table A1 generate *p* values of less than 10% for all variables except for the quadratic transformation of age distribution (z_2) These results suggest that most of our time series data support rejection of the null hypothesis of the conventional Dickey-Fuller unit root specification in favor of the alternative hypothesis of the Fourier specification. Following Chang, Lee and Chou (2012), a 10% significance level was used to select the lag order of $\tilde{S}_t$ in order to remove serial correlation in the residuals. The critical values of the *LM* test (that tests for the null hypothesis of the unit root property) for 10%, 5%, and 1% were obtained by the bootstrap method with 20,000 replications. Based on these critical values, the *LM* statistics ($\tau_{LM}(\hat{k})$) for all variables (except for the quadratic transformation of age distribution (z_2) displayed in Table A1) support rejection of the null hypothesis of the unit root property at the 10% (or stricter) significance level. The conventional ADF unit root testing statistics (generated in the same way as was used to select the lag order of $\tilde{S}_t$ for the *LM* statistics) for the quadratic transformation of age distribution (z_2) also supports rejection of the null hypothesis of the unit root property at the 1% significance level. These findings support the stationarity of all variables used to investigate the relationship between demographic structure and copayment policy effectiveness, so the statistical inferences obtained from the multiple linear regression model used to establish the nonlinear effect of age distribution on copayment policy effectiveness should be considered justified.

5. Results for the parametric age response function

Table A2 exhibits the empirical results for the parametric age response function specified by equation (A5). Two transformed variables of age distribution in the multiple linear regression model are significantly correlated with all measures of copayment policy effectiveness at 1% significance level. To portray the complete effects of demographic structure change on copayment policy effectiveness, we retrieved the estimated coefficients for shares of the total population in seven age-specific groups through the delta method, as demonstrated in the upper part of Table 3, and plotted their corresponding 90% confidence intervals in Fig 3(a)-(b).

Table A2 Results for the parametric age response function†

Variable	MRM		Cum-Max	
	Coefficient	t-value	Coefficient	t-value
z1	0.133	10.051***	1.164	2.900***
z2	-0.017	-10.058***	-0.163	-3.162***
CRH	-0.001	-1.694*	-0.017	-1.915*
ln(UR) $\times 10^{-2}$	0.024	0.913	0.736	1.163
ln(FLPR)	0.020	5.374***	0.772	6.472***
Constant	-0.118	-7.890***	-3.160	-6.791***

† "*", "**" , "***" represent 10%, 5%, and 1% significance levels, respectively. *t* values were computed by dividing the estimated coefficients by the Newey-West standard errors. CHR represents the contribution of the healthcare and social services sector to economic growth. UR and FLPR denote unemployment rate, and female labor participation rate, respectively.

References

Chang T, Lee CH, Chou PI. Is per capita real GDP stationary in five southeastern European countries? Fourier unit root test. Empirical Economics, 2012; 43: 1073-1082.

Enders W, Lee J. The flexible Fourier form and Dickey–Fuller type unit root tests. Economics letters, 2012; 117(1): 196-199.

MacKinnon JG. Numerical distribution functions for unit root and cointegration tests. Journal of Applied Econometrics, 1996; 11(6): 601-618.

Nakajima, J. Time-varying parameter VAR model with stochastic volatility: an overview of methodology and empirical applications. Monetary and Economic Studies, 2011; 29: 107-142.

Phillips, PCB., Perron, P. Testing for a unit root in time series regression. Biometrika, 1988;75, 335-346.
